# Supplementary material for: Saccharomyces boulardii (CNCM I-745) improves intestinal damage in sepsis by remodeling intestinal flora structure
Source: Front Cell Infect Microbiol. 2025 Jul 31;15:1602792. doi: 10.3389/fcimb.2025.1602792 (PMC12350329; doi:10.3389/fcimb.2025.1602792)
Supplement: Supplementary file 1 [file DataSheet1.zip › summary/summary/7_Differential_analysis/1_diff_abundance/6_Genus/Group.DW0_vs_CW0_vs_BW0_vs_AW0/Group.DW0_vs_CW0_vs_BW0_vs_AW0_diff_barplot.pdf]

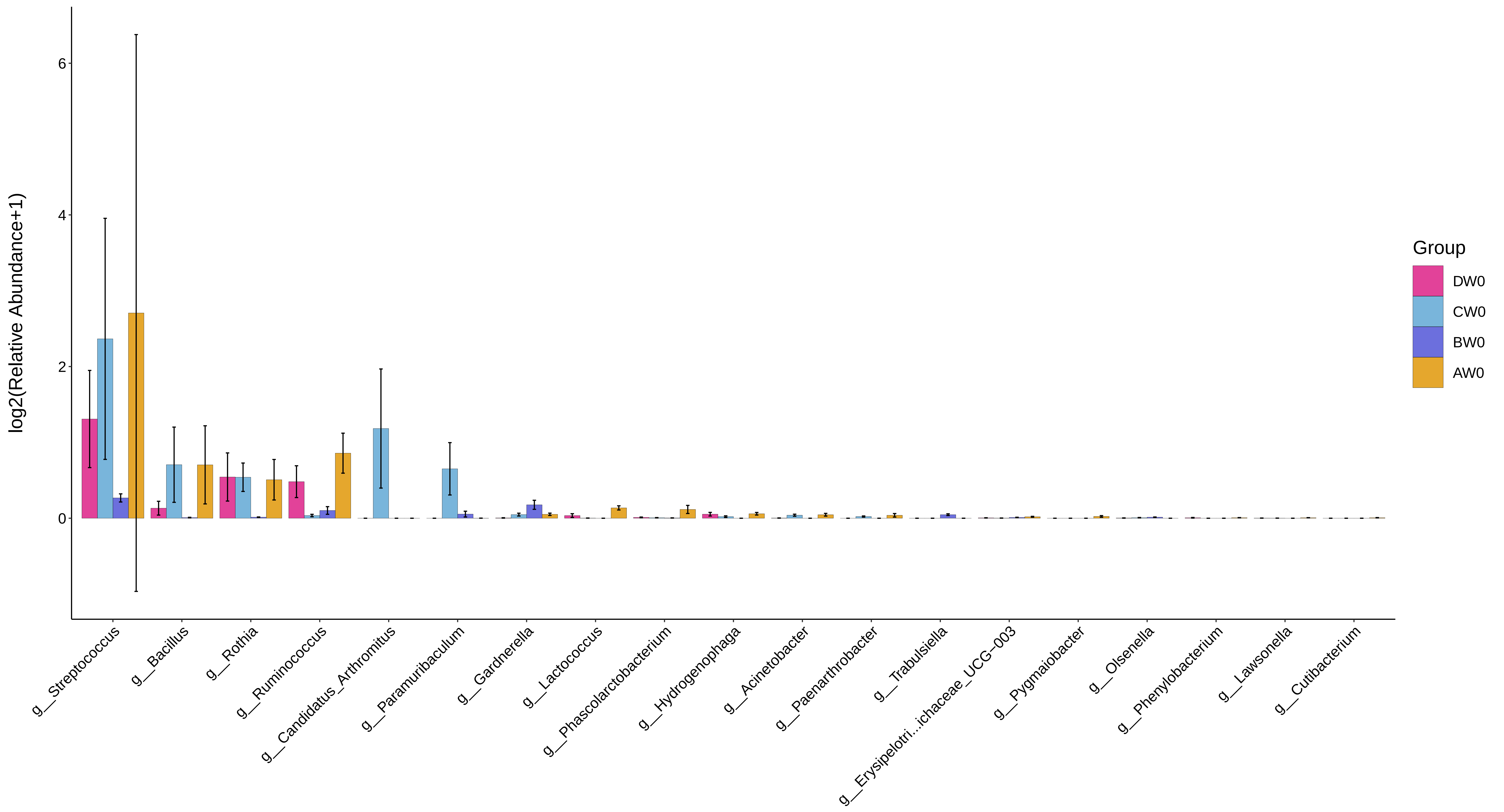

log2(Relative Abundance+1)
6
4
2
0
g__Streptococcus
g__Bacillus
g__Rothia
g__Ruminococcus
g__Candidatus_Arthromitus
g__Paramuribaculum
g__Gardnerella
g__Lactococcus
g__Phascolarctobacterium
g__Hydrogenophaga
g__Acinetobacter
g__Paenarthrobacter
g__Trabulsiella
g__Erysipelotri...ichaceae_UCG−003
g__Pygmaiobacter
g__Olsenella
g__Phenylobacterium
g__Lawsonella
g__Cutibacterium
Group
DW0
CW0
BW0
AW0
